# Successful Management of Recurrent Rectal Leiomyosarcoma: A Case Report

**DOI:** 10.1002/ccr3.71279

**Published:** 2025-10-30

**Authors:** Annmarie Butare, Warqaa Akram

**Affiliations:** ^1^ Department of Surgery Brody School of Medicine at East Carolina University Greenville North Carolina USA

**Keywords:** leiomyosarcoma, radiation, rectal, recurrence

## Abstract

Rectal leiomyosarcoma is rare and portends a poor prognosis with high recurrence rates. While no current gold‐standard treatment exists for rectal leiomyosarcoma, a disease‐free state can be successfully achieved with neoadjuvant radiation followed by total mesorectal excision.

## 1 Introduction

Rectal leiomyosarcoma is an exceedingly rare disease, comprising 0.1% of all forms of colorectal cancer [1]. Morphologically, leiomyosarcomas are firm, non‐encapsulated, well‐circumscribed tumors that originate from the muscularis propria or muscularis mucosa [2]. Intraluminal growth of the tumor eventually leads to mucosal ulceration and friability. More than 75% of patients are asymptomatic at the time of diagnosis; however, patients may present with altered bowel habits, bleeding, and pain [1]. The histologic features characteristic of leiomyosarcoma include sheets of densely packed spindle cells with varying degrees of necrosis, hemorrhage, and cystic degeneration. While sharing similar features with other stromal tumors, such as GIST, leiomyosarcoma can be differentiated based on immunohistochemical staining (i.e., Negative for CD117, DOG1, CD34, S100 and Positive for smooth muscle actin [SMA] and desmin) [1]. Factors associated with increased malignant potential include size greater than 5 cm, a high number of mitoses per high‐powered field, degree of cellular atypia, and the extent of tumor cell necrosis [3]. While the rectum is the least common site for GI leiomyosarcoma, it carries the poorest prognosis. Local recurrence and metastasis to the lungs or the liver contribute to a 5‐year survival rate of 50% [1, 2]. Current NCCN guidelines recommend oncologic resection as primary treatment, with the option for neoadjuvant systemic treatment only if proven high risk of metastatic disease or need for tumor size reduction for surgical resection [4, 5]. Given the rarity of this form of tumor and current debate on best practices, we present a case of a 71‐year‐old male who has achieved a disease‐free state following a multifaceted treatment approach.

## 2 Case History and Examination

The patient is a 71‐year‐old male who presented with unintentional weight loss of 17 pounds in 1 year, post‐prandial nausea, decreased appetite, and change in stool caliber, consistency, and frequency. His past medical history is significant for aortic valve replacement, chronic kidney disease, obesity, diabetes, hypertension, and hyperlipidemia. Personal and family history were negative for cancer. He never had a colonoscopy prior to this presentation. Digital rectal exam revealed a mobile, non‐bleeding, posterior mass palpated 6 cm from the anal verge. Sphincter tone was slightly diminished.

## 3 Differential Diagnosis

A change in bowel habits and weight loss is concerning for malignancy in this 71‐year‐old male. While there is no personal or family history of cancer, malignancy must be forefront in the differential diagnosis, particularly in the absence of a prior screening colonoscopy. Additional differential diagnoses include chronic constipation, rectal polyp, rectal abscess, hemorrhoids, anal fissure, and benign rectal mass such as fibroma or lipoma.

## 4 Investigation and Treatment

**FIGURE 1:**
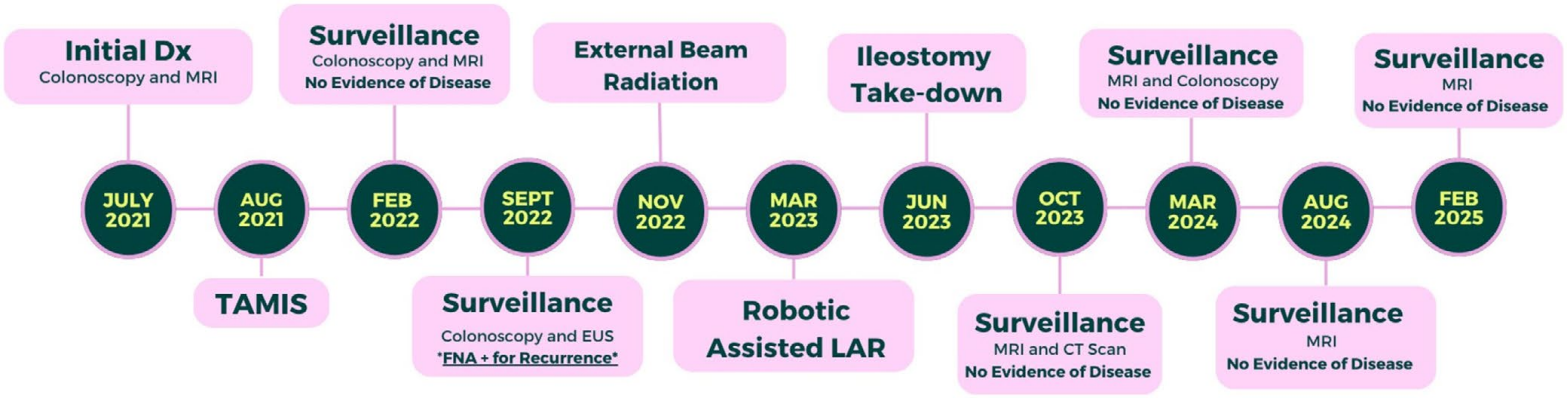
Timeline of events from diagnosis through current surveillance.

**FIGURE 2:**
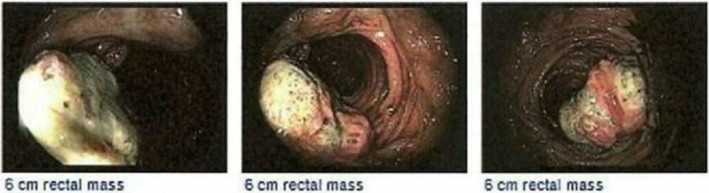
Endoscopic findings during initial diagnosis.

A timeline of events in this patient's treatment course is illustrated in Figure 1. A diagnostic colonoscopy identified a low rectal mass that was tattooed and biopsied (Figure 2). The tissue stained positive for Ki‐67 and SMA and negative for CD‐117, S‐100, and desmin. There was marked nuclear atypia and necrosis, with up to 24 mitotic figures per high power field. Initial diagnosis favored leiomyosarcoma. Staging cross‐sectional imaging included computed tomography (CT) scans and magnetic resonance imaging (MRI), which showed a 4.5 × 3.4 cm polypoid mass without evidence of perirectal invasion or distant metastasis. The mass was located 7.4 cm from the anal verge and graded T1/T2.

**FIGURE 3:**
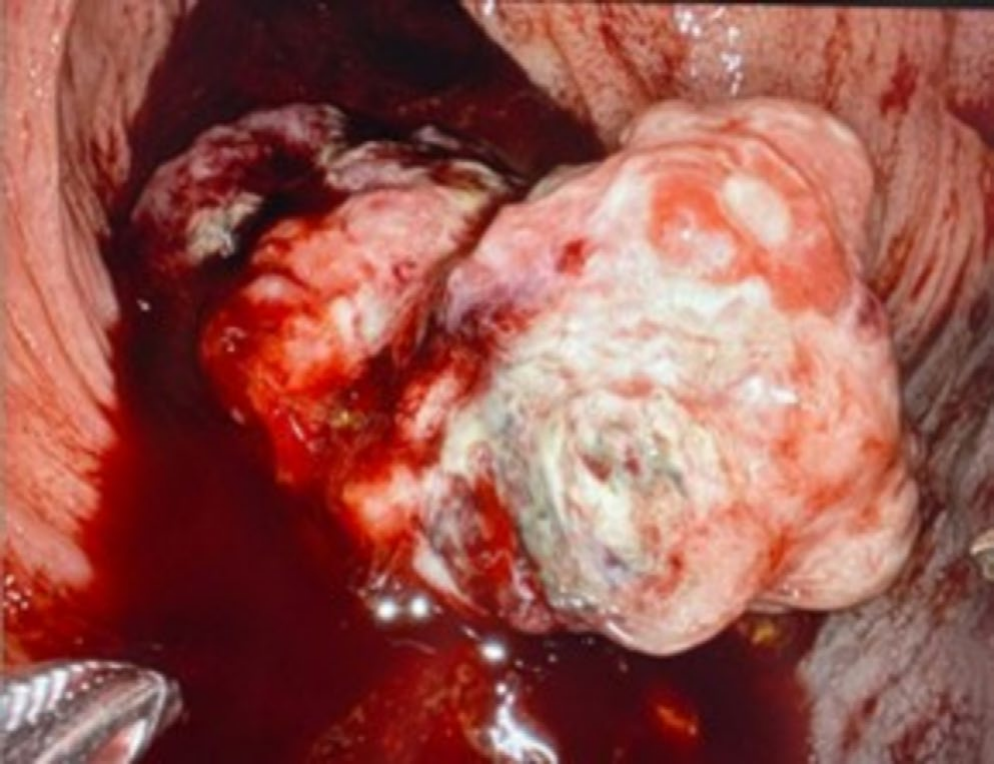
Intra‐operative view of rectal mass during TAMIS.

**FIGURE 4:**
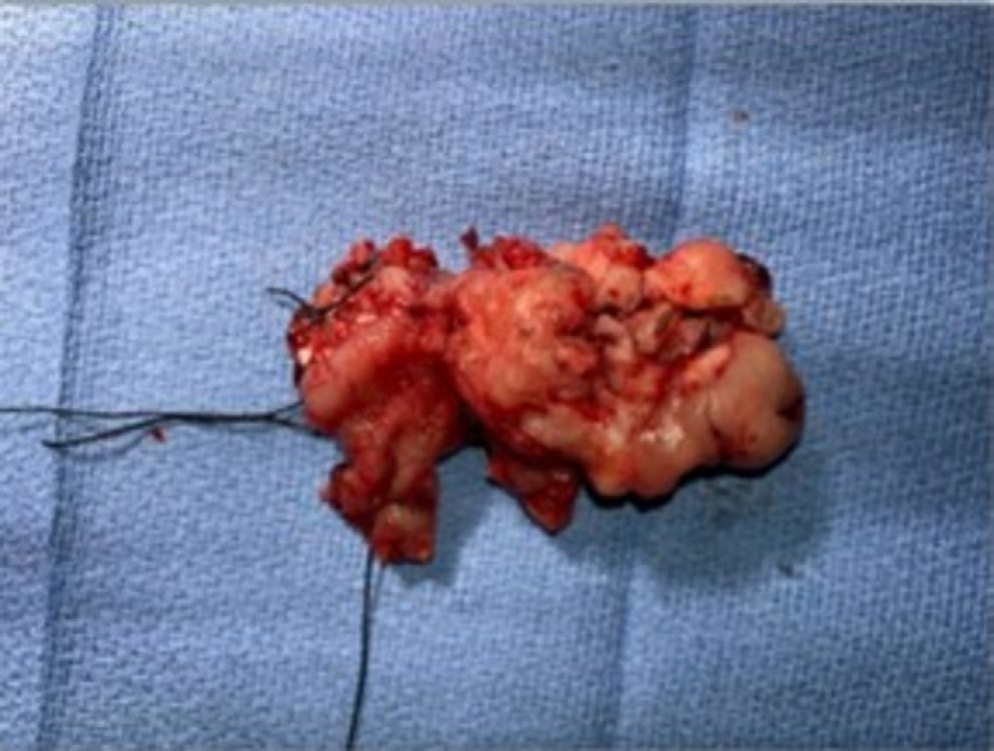
Gross examination of resected tumor following transanal minimally invasive surgery (TAMIS).

This case was discussed at a Multidisciplinary Tumor Board at a tertiary cancer center. Given the rarity of lymph node spread in soft tissue sarcoma and diagnostic imaging to confirm tumor confinement within the rectal wall, the decision was made to proceed with robotic‐assisted transanal resection. Intra‐operatively, the mass was noted to be friable and pedunculated (Figure 3). It was excised completely with gross margins (Figure 4). Final pathology resulted as Grade 3 Leiomyosarcoma, measuring 5.7 × 4.6 × 2.5 cm with a FNCLCC (French Federation of Cancer Centers Sarcoma Group) score of 6/8 (2/3 for tumor differentiation, 3/3 for mitotic activity with 20 mitotic figures per high power field, and 1/2 for necrosis) [6]. All resection margins were negative, with the closest margin being 0.3 cm. As surgical resection is considered the mainstay of treatment and there is no clear role for chemotherapy, his post‐operative management consisted of high frequency surveillance, with MRI at 6‐month intervals. At 3, 6, and 9 months post‐op, the patient endorsed symptom improvement, including improved appetite, weight gain, and return of normal bowel function. Physical exam and MRI revealed no concerning findings in the initial 9 months following surgery.

**FIGURE 5:**
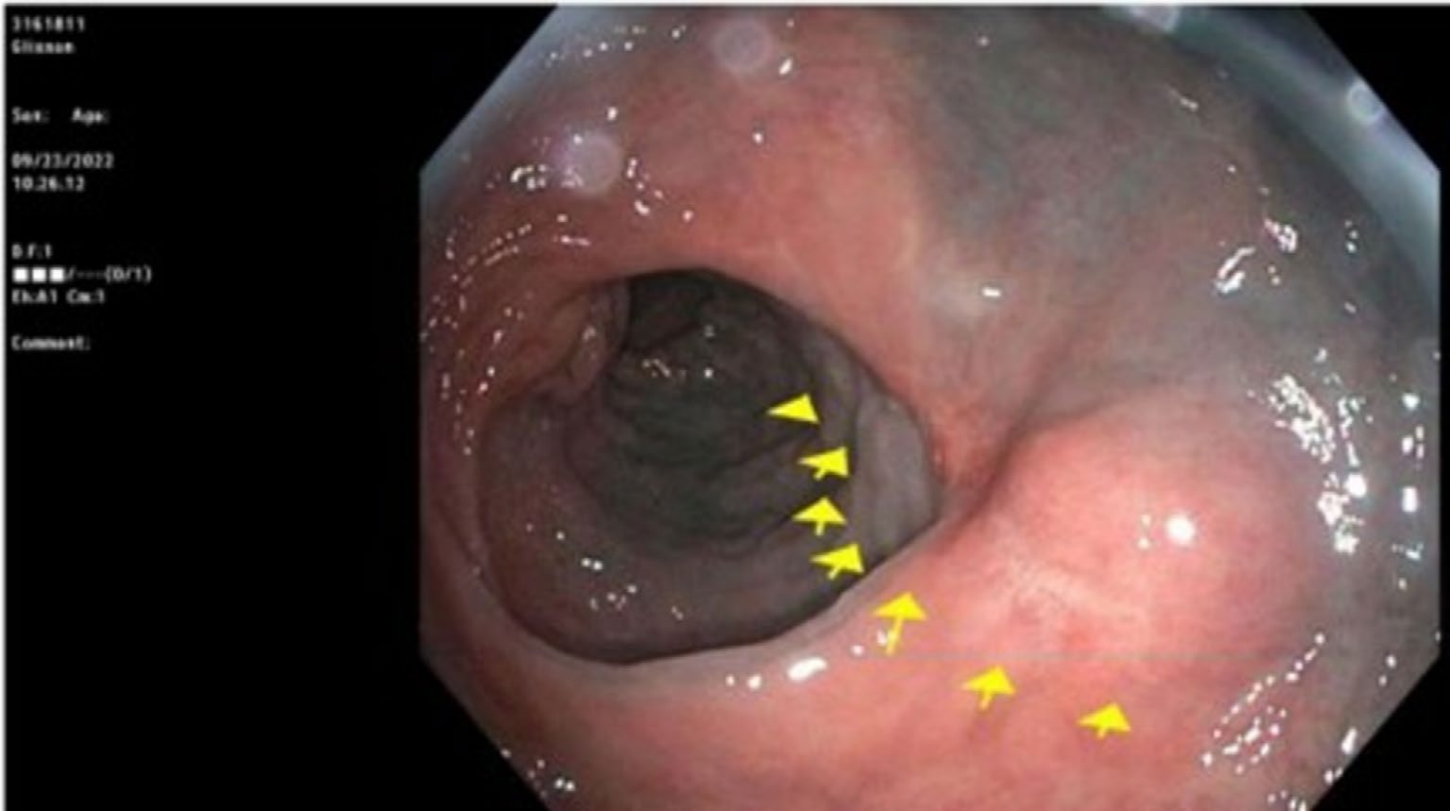
Endoscopic findings of recurrence of tumor at site of previous scar.

**FIGURE 6:**
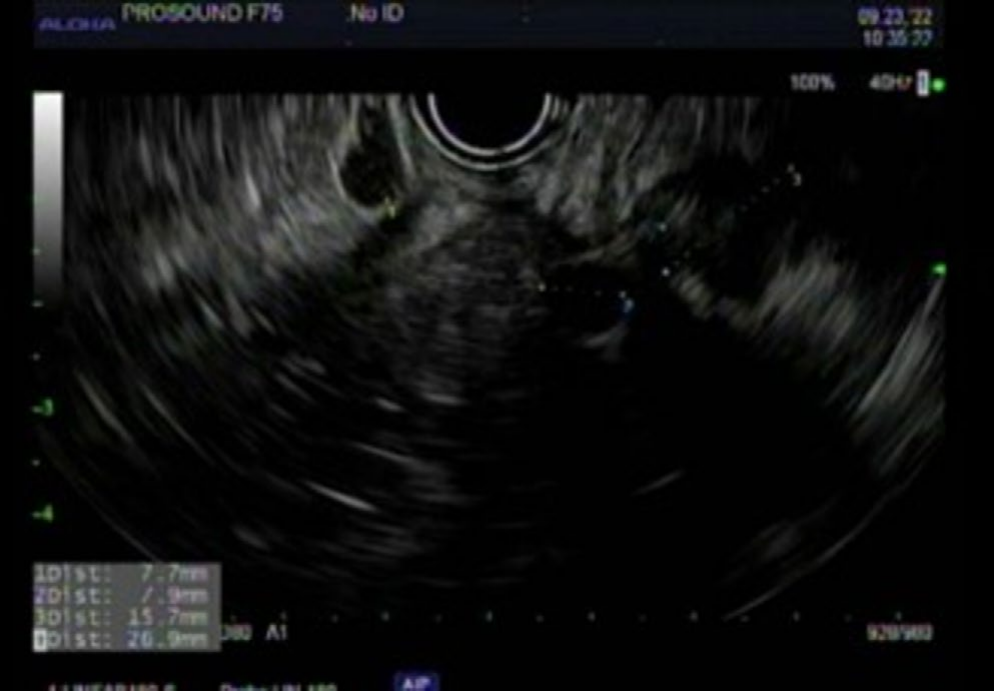
Endoscopic ultrasound findings of recurrence of tumor at site of previous scar.

**FIGURE 7:**
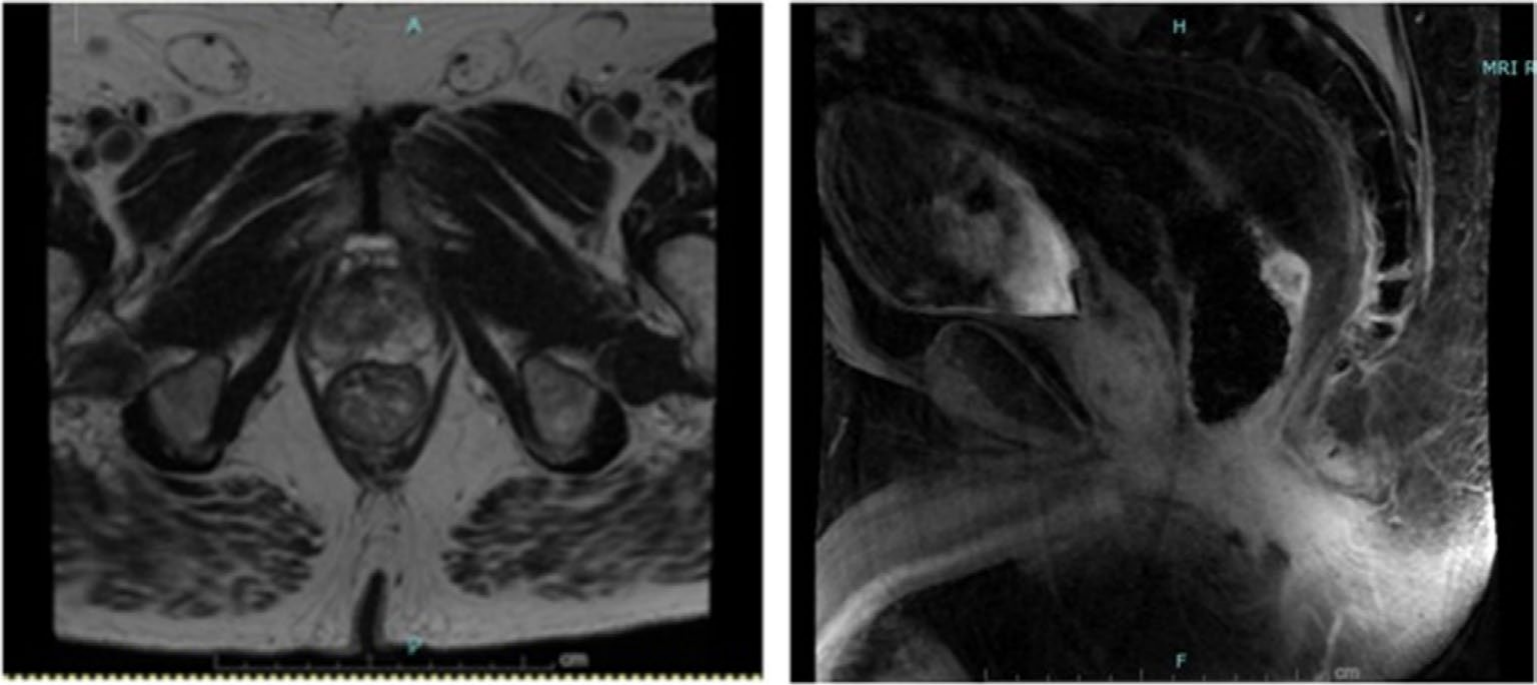
MRI image of tumor recurrence.

**FIGURE 8:**
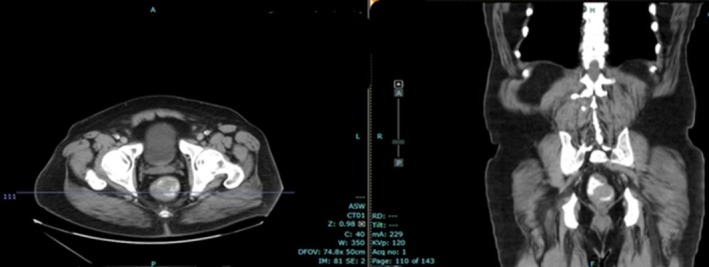
CT scan image of recurrence.

One year following initial resection, endoscopic examination with ultrasound was completed for surveillance. Three sub‐epithelial lesions were identified along the surgical scar (Figures 5 and 6). All lesions appeared to have originated from muscularis propria and measured 7–8 mm. The largest lesion was biopsied with FNA, showing recurrence of leiomyosarcoma with increased mitotic activity, increased atypia, and immunoreactivity for SMA and desmin. No suspicious lymph nodes were identified. Representative MRI and CT scan images are seen in Figures 7 and 8, respectively. The case was again discussed at the Multidisciplinary Tumor Board, with the recommendation to proceed with neoadjuvant radiation followed by resection with total mesorectal excision (TME), given the short interval of recurrence. He completed 5 weeks of targeted external beam radiation and returned to the operating room approximately 19 months after his initial resection for robotic‐assisted low anterior resection with diverting loop ileostomy.

## 5 Outcome

Final pathology revealed a 2.5 cm Grade 1 leiomyosarcoma, with a FNCLCC score 3/8 (2/3 for tumor differentiation, 1/3 for mitotic rate with seven figures per high power field, and 0/2 for necrosis). The tumor invaded perirectal tissue, but all resection margins were negative for tumor, with the closest margin being 1.5 cm. Nine lymph nodes were examined and found to be negative for tumor. The final pathologic stage was rypT1N0. He has followed up for surveillance every 3 months with imaging for 2 years since his formal resection. He is currently in a disease‐free state. He will continue surveillance every 6 months for an additional 2–3 years, in accordance with NCCN guidelines [6].

## 6 Discussion

The mainstay of treatment for rectal leiomyosarcoma has been surgical resection, as current literature fails to show demonstrative improvement with chemotherapy or radiation. The goal of surgical excision is to remove all diseased tissue, with less emphasis on the removal of lymphatic tissue, as this tumor spreads via direct invasion rather than lymphatic spread [3]. Some authors argue for local excision for tumors < 2.5 cm that are limited to the bowel wall based on preoperative imaging [2]. In other cases, radiation has been used as an adjunct to preserve sphincter tone or allow for a more limited resection [2]. Regardless of the type of resection, recurrence rates of rectal leiomyosarcoma are exceedingly high, ranging from 25% following abdominoperineal resection to 88% following local excision [4]. The patient in this case presented with a low rectal mass, invading the submucosa and muscularis propria, without threatened mesorectum. There is a lack of evidence to support the use of neoadjuvant chemotherapy or radiation, so the decision was made to proceed with local resection, and the tumor was removed with negative margins. Chemotherapy has classically been limited to patients who have unresectable or metastatic disease, and more research is needed to support the use or avoidance of chemotherapy in rectal leiomyosarcoma [7, 8]. One retrospective study by Tseng et al. evaluated the effect of 2–4 cycles of anthracycline‐based chemotherapy [9]. In a cohort of 158 patients, no patient had a complete response, and only 23% had a partial response. While all patients ultimately underwent a complete resection, there was worse overall survival noted in patients who had disease progression while on chemotherapy [9]. These findings, among others, emphasize the resistance of rectal leiomyosarcoma to chemotherapy and occasionally worse survival rates among patients who receive chemotherapy for rectal leiomyosarcoma [8].

Neoadjuvant radiation is the standard of care for extremity leiomyosarcoma; however, its benefits have not been proven for abdominal leiomyosarcoma [10]. One systematic review describes the dichotomy of results with neoadjuvant radiation, as one study found reduced risk of local recurrence and increased odds of R0 resection, while another study found no impact of neoadjuvant radiation on overall survival [8]. Furthermore, Nassif et al. found a 75% recurrence rate among patients who received adjuvant radiation [11].

The evidence for and against the use of neoadjuvant chemotherapy or radiation has been limited to retrospective reviews and case studies until the STRASS trial was completed in 2020 [12]. In this phase 3 randomized controlled trial, comparing preoperative radiation followed by surgery and surgery alone found no significant difference in recurrence‐free survival. In fact, there was a significant increase in adverse effects of therapy in the radiation group. The authors concluded that neoadjuvant radiation therapy should not be considered standard of care [12]. A similar randomized controlled trial entitled STRASS2 is currently in the recruitment phase. This phase 3 trial compared neoadjuvant chemotherapy followed by surgery to surgery alone [13]. Until this trial and further high‐quality randomized controlled trials are published, surgeons are left with limited low‐ and moderate‐level evidence with which to plan for their patients.

Following local resection and pathologic review, this patient was deemed to be at high risk for recurrence due to a high mitotic rate and degree of tumor necrosis. However, considering the potential morbidity of TME and lack of evidence to support recurrence‐free survival after TME, the decision was made to undergo close surveillance rather than further wide excision based on tumor pathology factors. Recurrent rectal sarcoma tends to be more poorly differentiated, more anaplastic, and have a higher mitotic rate than the primary tumor [4]. The patients who have been reported in the literature to have had recurrence have had much poorer outcomes, with death most often occurring within 1 year of recurrence. This supports the decision for more aggressive treatment for this patient's recurrence, with neoadjuvant radiation and TME.

Rectal leiomyosarcoma is a rare disease, with limited available data in the literature to support management. This case report describes a classic presentation of rectal leiomyosarcoma, initially treated with local excision, with recurrence successfully managed with neoadjuvant radiation and TME to achieve a disease‐free state. This case demonstrates the decision‐making process involved in balancing the benefit of avoiding an extensive, potentially morbid resection, with a known risk of recurrence in both local resection and extensive resection, using available evidence in the literature and shared decision‐making with a Multidisciplinary Tumor Board.

## Author Contributions

**Annmarie Butare:** conceptualization, data curation, investigation, methodology, resources, writing – original draft, writing – review and editing. **Warqaa Akram:** conceptualization, data curation, investigation, methodology, project administration, supervision, writing – review and editing.

## Disclosure

The authors have nothing to report.

## Consent

Written informed consent was obtained from the patient to publish this report in accordance with the journal's patient consent policy.

## Conflicts of Interest

The authors declare no conflicts of interest.

## Data Availability

Data sharing not applicable to this article as no datasets were generated or analyzed during the current study.
